# SG-APSIC1087: Transcriptome meta-analysis revealed concordant molecular signatures between acne skin and PM2.5-treated in vitro skin models

**DOI:** 10.1017/ash.2023.41

**Published:** 2023-03-16

**Authors:** Xuelan Gu, Xiao Cui, Hong Zhang, Grace Mi

**Affiliations:** Unilever, China; Unilever Research and Development Center, Shanghai, China; Unilever Research and Development Center, Shanghai, China; Unilever Research and Development Center, Shanghai, China

## Abstract

**Objectives:** Cohort and epidemiology studies have previously revealed potential associations between air pollution exposure and acne vulgaris. However, the molecular mechanisms that drive these associations are not currently well understood. In this study, we compared the molecular signatures of acne and PM2.5-exposed skin to infer whether common underlying biological mechanisms exist. **Methods:** Acne microarray data sets were downloaded from GEO. RMAExpress was used for microarray normalization, and TMeV was used to identify differential expressed genes (DEGs). A random-effects model in MetaVolcanoR was used to determine fold changes and P values. DEGs of PM2.5-exposed skin-cell models were obtained from the literature. DEGs were compared using GeneOverlap and a custom R script. Analyses of pathways, upstream regulators, and causal networks were conducted using ingenuity pathway analysis (IPA). **Results:** The molecular signatures of acne skin and the effect of PM2.5 on skin in vitro were compared at 3 levels: (1) gene expression, (2) pathway activity, and (3) upstream regulators. Significant concordant overlaps of both upregulated (*P* < 3e-23) and downregulated DEGs (P< .005) were observed in acne skin and PM2.5-exposed keratinocytes. However, for the PM2.5-exposed 3D skin model, significant overlap with acne skin was only observed for upregulated DEGs (*P* < 8e-14). Fold changes of DEGs in both acne and PM2.5-exposed data sets showed significant correlation (Pearson correlation coefficient > 0.6; *P* < .001). An IPA analysis identified 13 pathways commonly enriched in acne and PM2.5 data sets, including IL17, IL6, Toll receptor PPAR, LXR–RXR, and acute-phase response pathways. Common upstream regulators were further identified including TNFα, NFκB, CAMP, AhR, and IL17A. Finally, causal network analysis revealed several potential hub regulators shared in acne pathogenesis and PM2.5-exposed skin, including HIF1α, TNF, IL1α, and CCL5. **Conclusions:** Our analysis revealed significant concordant molecular signatures between acne and PM2.5-exposed skin. Biological insights from this study offer clues that build the causal links between air pollution and acne pathogenesis.

